# Early Morning Functional Impairments in Stimulant-Treated Children with Attention-Deficit/Hyperactivity Disorder Versus Controls: Impact on the Family

**DOI:** 10.1089/cap.2016.0164

**Published:** 2017-10-01

**Authors:** Stephen V. Faraone, Russell J. Schachar, Russell A. Barkley, Rick Nullmeier, F. Randy Sallee

**Affiliations:** ^1^ Departments of Psychiatry and Neuroscience and Physiology, SUNY Upstate Medical University, Syracuse, New York.; ^2^ Department of Psychiatry and Research Institute, The Hospital for Sick Children, University of Toronto, Toronto, Canada.; ^3^ Department of Psychiatry, Medical University of South Carolina, Charleston, South Carolina.; ^4^ Ironshore Pharmaceuticals & Development, Inc., Camana Bay, Grand Cayman, Cayman Islands.

**Keywords:** ADHD, morning, family, siblings, parents, functioning

## Abstract

***Objective:*** Children with attention-deficit/hyperactivity disorder (ADHD) frequently manifest early morning functional (EMF) impairments before school. We conducted a quantitative research survey to assess the impact of these EMF impairments on the family unit (caregiver, spouse/partner, and siblings).

***Study Design:*** We developed an online survey questionnaire to collect data from 300 primary caregivers of children with ADHD and 50 primary caregivers of children who did not have ADHD.

***Results:*** Although the ADHD children we surveyed were currently treated with stable doses of stimulants as their primary ADHD medication for at least 3 months, their parents reported high levels of EMF impairments in the child, which had a substantial negative effect on the emotional well-being of parents, on parents' functioning during the early morning routine, and on the level of conflict with siblings. The impact of EMF impairments on family functioning was mediated by the severity of the index child's impairments.

***Conclusions:*** EMF impairments exert a pervasive and significantly negative emotional and functional burden on not only the primary caregiver but also on the spouse/partner and siblings. This work suggests that adequate ADHD symptom control during the early morning period may be an unmet need for school-age children with ADHD being treated with stimulants. More work is needed to confirm this finding and determine the degree to which symptom control at other times of day is also an unmet need.

## Introduction

Attention-deficit/hyperactivity disorder (ADHD) is a common, persistent, and impairing psychiatric disorder affecting many children aged 3–17 years worldwide (Faraone et al. 2015a). Although much is known about how ADHD impacts patients and their families (Faraone et al. 2015a), little is known about the effects of ADHD during particular times of the day, especially on early morning functioning (EMF).

The EMF of children with ADHD, especially on school days, deserves special attention for several reasons. Between waking and arriving at school, children must appropriately sequence and complete a series of complex behaviors (e.g., dressing, eating, self-hygiene/brushing teeth, and gathering school books) in the context of other family members who may also be engaged in the same or similar goal-directed activities. Completing these behaviors requires time management, working memory, and self-regulation skills as well as social skills and cooperation that are frequently impaired by ADHD symptoms (Whalen et al. 2006). When children fail to efficiently complete their morning routine, it puts them at risk for being late to school and forgetting to take homework and other materials to school. These issues may lead to academic and social difficulties.

Inadequate control of ADHD symptoms during the early morning routine before school can also be significantly disruptive to siblings and parents who must also be prepared and depart the home for their own scheduled activities in a timely manner.

A study by Barkley and Cunningham (1979) showed that ADHD impaired early morning organization, self-care, preparation for the school day, and transportation to school. Whalen et al. (2006) showed that children's ADHD symptoms led to less effective parenting behaviors, especially before school. Sallee (2015) surveyed 201 primary caregivers of youth with ADHD treated with stable doses of stimulant medication. Despite being maintained on stimulant medications, 75% of the caregivers rated their child's early morning routine before school as a period associated with moderate-to-severe symptoms of the disorder and related functional impairments. Many caregivers also experienced negative emotions related to their child's early morning impairments. The caregivers experienced stress while getting their child ready for school as well. About half said that these symptoms and impairments were harmful to the parent–child relationship. The authors concluded that early morning ADHD symptoms and EMF impairments were inadequately controlled for many youth with ADHD treated with stable morning doses of stimulant medications.

Given that EMF impairments may produce downstream adverse impacts on the entire day for the affected child as well as other family members, several studies assessed the efficacy of ADHD treatments for EMF impairments. Two placebo-controlled clinical trials assessed EMF impairments using the Before-School Functioning Questionnaire (BSFQ), which has documented reliability, internal homogeneity, and concurrent validity (Faraone et al. 2015b). Wilens et al. (2013) randomized ADHD youth to receive either continued stimulant treatment plus guanfacine extended release (GXR) in the morning (GXR AM) or evening (GXR PM), or continued stimulant treatment plus placebo. Parent-rated BSFQ scores indicated that EMF impairments improved with both GXR AM and PM. A crossover study of ADHD youth compared the methylphenidate transdermal system (MTS) with a placebo transdermal system (PTS) (Wilens et al. 2010). Compared to the PTS, MTS significantly reduced the investigator-rated BSFQ total score, but not the child self-rated BSFQ total score. The Daily Parent Rating of Evening and Morning Behavior (DPREMB) (Faraone et al. 2015a) was used by Michelson et al. (2002) in a double-blind study of atomoxetine treatment. Atomoxetine was not better than placebo for improving four early morning behaviors reported by parents. In contrast, two studies using a revised version of the same scale (DPREMB-R) reported greater improvements in EMF for atomoxetine versus placebo in randomized controlled clinical trials (Sutton et al. 2003; Kelsey et al. 2004), and two trials showed that, compared with stimulant treatment, atomoxetine was more effective for treating EMF impairments (Sangal et al. 2006; Whalen, et al. 2010).

The few data available on EMF and ADHD suggest that it is a considerable source of impairment for children with ADHD as well as stress within their families, and current pharmacotherapies show variable efficacy. However, no prior study has assessed EMF impairments and its effects on both the stimulant-treated child with ADHD and their family using a controlled study. Such a study of stimulant-treated children is needed to determine if EMF impairments are an unmet need for the pharmacotherapy of ADHD. We did not include children treated with nonstimulants due to cost considerations and the fact that only a small minority of ADHD youth are treated with nonstimulants as their primary ADHD medication.

To fill this gap in the literature, we conducted a survey of primary caregivers of children with and without ADHD. We had several goals: (1) to assess the nature and severity of EMF impairments associated with stimulant-treated ADHD and (2) to assess the prevalence, frequency, and impact of EMF impairments on the caregiver, spouse/partner, and siblings of stimulant-treated ADHD children and adolescents. We hypothesized that compared with families not having children with ADHD, those having children with ADHD, who expressed at least mild ADHD symptoms in the morning, would, despite being treated with stimulants, show higher levels of EMF impairments and these impairments would have deleterious effects on the family. We also tested the hypothesis that the impact on the family would be mediated by the severity of the EMF impairments.

## Methods

Survey participants were 300 caregivers of children diagnosed with ADHD and 50 caregivers of children not diagnosed with ADHD. Potential survey participants were drawn from the Lightspeed GMI US Panel (*N* = 1,269,000). The Lightspeed GMI panel is constructed so that its consented panel members are generally representative of the U.S. population in terms of age, gender, income, ethnicity, geography, employment, and educational levels in the household (see Appendix). GMI collects a wide range of valuable consumer information about habits, characteristics, behaviors, and medical conditions that aid in survey targeting. To improve survey productivity, our panel sample for parents of ADHD children was initially drawn from the subset of GMI US panel members who had previously identified that they had a child with ADHD aged 6–17 years (targeted sample, *n* = 17,130). Once that subset was exhausted, survey invitations were sent to the subset of GMI US panelists with children aged 6–17 years (*n* = 253,800). Lightspeed GMI also provided the survey programming and online hosting for the project.

As an incentive for participation, those who completed the survey were awarded points as participation incentives. The number of points awarded is based on a proprietary formula that factors in survey length and difficulty of recruiting. A longer survey has a higher point value incentive than a shorter one. A survey with a difficult-to-recruit audience awards more points than an easier-to-recruit survey. After accumulating points, members are able to redeem them for items within Lightspeed GMI's rewards catalog. Examples of items panel members can redeem points for include the following: PayPal and Amazon e-certificates, gift cards, vouchers, cash, electronics, and home and personal care products.

Inclusion in the ADHD group required four criteria: (1) being self-identified as the primary caregiver of an ADHD child aged 6 to 17 years; (2) the child with ADHD was taking stimulant medication as his or her primary ADHD medication; (3) he or she was taking a stable dose of their primary stimulant medication for 3 months or more; and (4) the caregiver rated that the severity of the child's ADHD symptoms throughout the entire day and during the early morning routine, as two or more on a scale of 1 (no symptoms) to 10 (significant symptoms). We selected ADHD children showing at least mild evidence of ADHD in the morning because our goal was to determine if the expression of ADHD symptoms in the morning was associated with impairments in stimulant-treated children.

The study was conducted in April of 2016. Caregiver respondents were blinded as to the research sponsor. The survey was conducted in accordance with and adherence to the Marketing Research Association Code of Standards. Potential respondents were invited to participate through an email message. The survey invitation did not specifically refer to ADHD or/and EMF; so it did not bias participation toward families struggling with this domain of problems. The exact wording of the invitation was, “Today we are conducting a marketing research study concerning healthcare for your family. Your opinions are important to us. Please continue with the survey.” The survey, which was administered as an online questionnaire, required about 20 minutes for completion. It did not contain any option for any of the 18 standard types of PHI data to be collected and thus did not require IRB approval. Surveys were completed voluntarily and anonymously.

For the ADHD families, if there was more than one child with ADHD in the household, respondents were instructed to select the ADHD child who had the most severe ADHD symptoms. For the non-ADHD sample, respondents were instructed to select the child whose birthday was next. We refer to these selected children as the “index” children. The questions asked to caregivers of ADHD and non-ADHD youth were slightly different. For example, regarding concerns for safety, the former were asked the following question: “How often do your child's inadequately controlled ADHD symptoms during the early morning routine (before school) cause you concern for their safety and well-being in the home?” and the latter were asked the following question: “How often does your child's behavior during the early morning routine (before school) cause you concern for their safety and well-being in the home?”

We compared ADHD and non-ADHD families using logistic regression with ADHD status as the outcome and the demographic and family impact measures as independent variables. We first tested for demographic differences and included significant demographic predictors as covariates in all models. Because the family impact measures are conceptual outcomes, a more standard approach would have been to use these outcomes as dependent variables. However, doing so creates analytic problems due to the extreme nonnormality of the data and the strong assumptions we would need to analyze ordinal data. Logistic regression requires minimal assumptions and provides a valid method of establishing the statistical significance of the association.

To test the hypothesis that the impact on the families of ADHD youth was mediated by the severity of the EMF impairments, we included the severity of EMF impairments in the index child as a covariate in models testing for the association of ADHD family status and family impact. Our measure of severity was the answer to the following question: “On a scale from 1 to 10, where 1 means “Mildly Impaired” and 10 means “Very Severely Impaired”, how severe is that child's functional impairment (or difficulty to function) during the early morning routine?.”

## Results

The families with and without ADHD children did not differ in age of the index child (11.6 vs. 11.9; z = 0.5, *p* = 0.6), or relationship of the caregiver to the child (X^2^[3] = 1.6, *p* = 0.7). The index children in the ADHD group were more likely to be male (68% vs. 44%; z = 3.2, *p* = 0.001). Thus, all the following analyses are statistically corrected for sex of the index child. Forty-six percent of the ADHD youth were taking an amphetamine formulation and 54% were taking a methylphenidate formulation. Twenty-seven percent had been taking their medication for 3 to 6 months; 21% for 6 months to a year; 21% for one to 2 years; and 31% for more than 2 years.

### EMF impairments among ADHD children

Overall, 77% of caregivers rated the severity of EMF impairment in their child with ADHD as moderate-to-severe (severity rating of 5–10 on a 10-point severity scale). On the same severity scale from 1 to 10, where 1 means “Mildly Impaired” and 10 means “Very Severely Impaired,” ADHD children were rated as having higher mean levels of EMF impairment compared with controls (6.2 vs. 1.5; z = 6.7, *p* < 0.001). This shows that ADHD children who express some ADHD symptoms in the morning are at risk for EMF impairments. Consistent with this, the median number of school days each week that the child had EMF impairments was greater for youth with ADHD (4 days per week vs. 1 day per week; z = 5.8, *p* < 0.001).

**FIG. 1.**
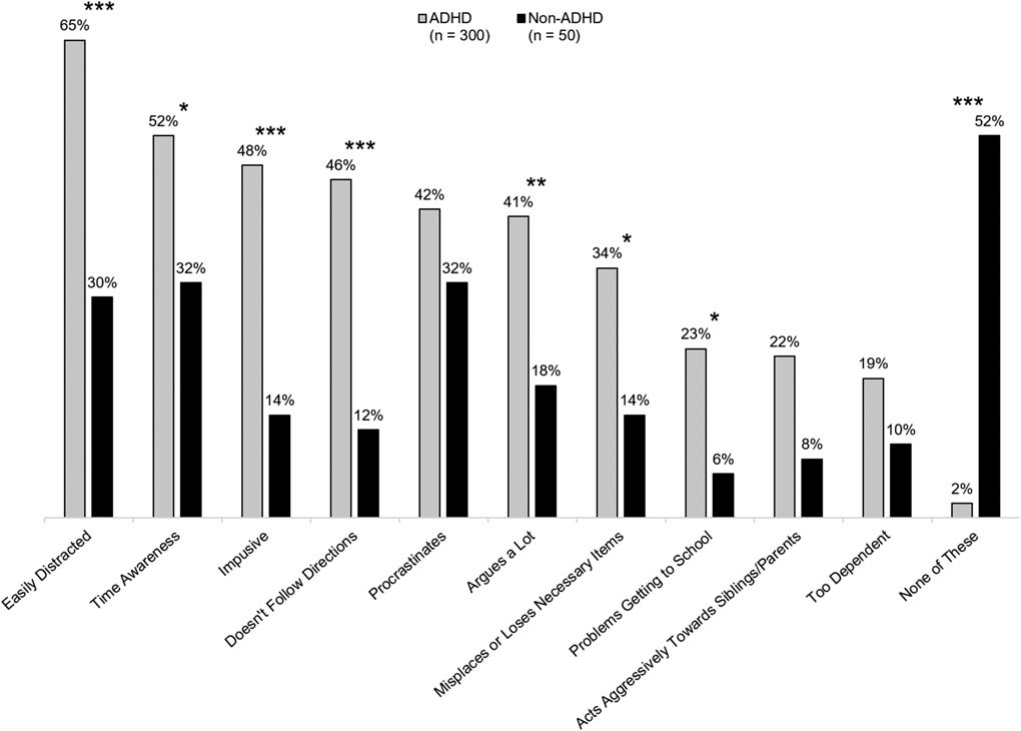
ADHD child's maladaptive behaviors and EMF impairments occurring “frequently.” Caregivers were given a list of EMF behaviors and asked to indicate which ones occurred “frequently.” *p < 0.05, **p < 0.01, and ***p < 0.001 vs. non-ADHD. ADHD, attention-deficit/hyperactivity disorder; EMF, early morning functioning.

We asked parents about 10 maladaptive behaviors occurring during the EMF period. Seven of these impairments were significantly more common among youth with ADHD compared with those without ADHD, and only 2% of youth with ADHD demonstrated none of these frequent maladaptive behaviors compared with 52% of non-ADHD youth (Fig. 1). Seventy-eight percent of the parents of ADHD youth had discussed the issue of EMF impairments with their doctor.

We asked the parents of the children with ADHD if they, or another adult in the household, ever woke their child with ADHD up earlier than their normal waking time to administer ADHD medication and then let them go back to sleep, so that the medication could provide more effective ADHD symptom control in the early morning. Fifty-seven percent indicated that they had used this strategy a median of 4 days per week during the school year. Seventy-eight percent of those who used this strategy indicated that its impact was either very positive or somewhat positive.

### Impact of EMF impairments on parents and siblings

**FIG. 2.**
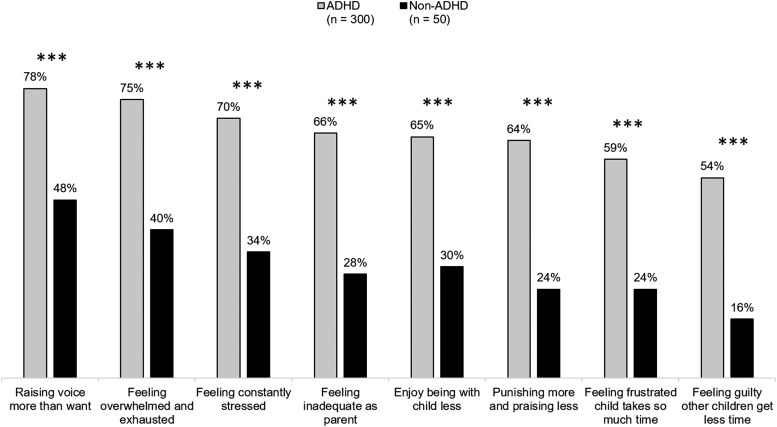
Parent feelings toward their child's impaired EMF occurring “sometimes” or “often.” Caregivers rated behaviors on a four-point scale: “never occurs,” “rarely occurs,” “sometimes occurs,” and “often occurs.” ***p < 0.001 vs. non-ADHD. ADHD, attention-deficit/hyperactivity disorder; EMF, early morning functioning.

As shown in Figure 2, the child's EMF impairments had a substantial and statistically significant negative impact on the emotional well-being of the caregivers of youth with ADHD. In response to EMF impairments, the caregivers of youth with ADHD were significantly more likely than caregivers of non-ADHD youth to report raising their voice more often, and feeling overwhelmed and exhausted, constantly stressed, inadequate as a parent, frustrated their child with ADHD consumed all their time, and guilty they were neglecting their other children.

Compared with caregivers of youth without ADHD, the caregivers of youth having ADHD were more likely to report that EMF impairments led to more stress from sibling conflict, greater disruption of the child's breakfast, greater disruption of the caregiver's morning routine, and a greater likelihood of being late for their own morning activities (all *p'*s < 0.001). We found similar results when assessing the effects of the child's EMF impairments on the spouses/partners of the caregivers. During the early morning period, the caregivers of youth with ADHD reported significantly more conflict with their spouses/partners, more disruption of their spouse's/partner's early morning routine, and that the child's EMF impairments kept the spouses/partners from being on time (all *p'*s < 0.01).

**FIG. 3.**
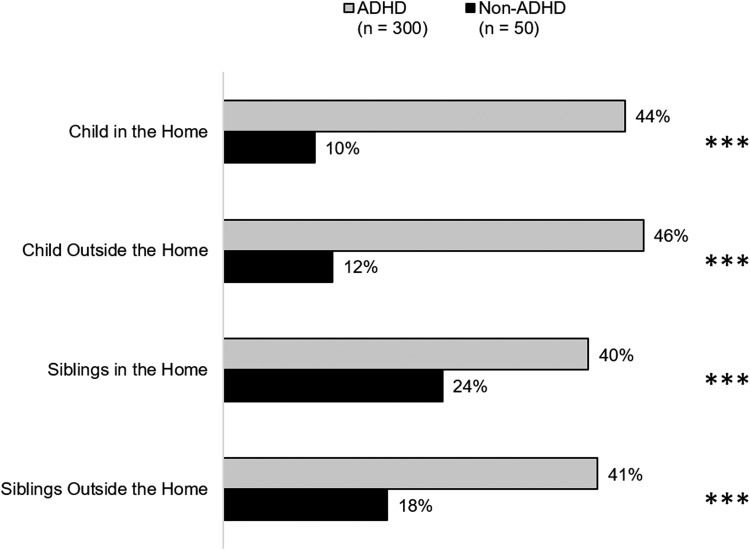
Parent concern about their child's/sibling's safety occurring “often” or “very often.” Caregivers rated behaviors on a five-point scale: “never,” “rarely,” “sometimes,” “often,” and “very often.” ***p < 0.001 vs. non-ADHD. Note: 258 ADHD families had siblings and 34 non-ADHD families had siblings. ADHD, attention-deficit/hyperactivity disorder.

Parents were asked how often EMF impairments caused them concern for the index child's safety and well-being inside and outside the home. The same question was also asked if such ADHD-related EMF of the index child also affected the safety and well-being of the siblings. As Figure 3 shows, concerns about index child and sibling safety were significantly and substantially higher among the parents of children with ADHD (all *p'*s < 0.001).

We also assessed the impact of the index child's EMF on the morning routines of their siblings. Compared with the parents not having an ADHD child, those with an ADHD child were more likely to report significant or very significant disruptions of the siblings' morning routines (42% vs. 18%, *p* < 0.001), disruptions causing siblings to be late (*p* < 0.001), disruption of the family breakfast (*p* < 0.001), and conflict with siblings (*p* < 0.05). Fifty-two percent of parents with ADHD children reported that sibling complaints about the index child's EMF impairments or disruption of the sibling's morning routine occurred often or very often compared with 18% of control families (*p* < 0.001).

### Mediation of impact on family by severity of EMF impairments in the child

We added the severity of the EMF impairments in the index child as a covariate in all the models testing for the association of ADHD family status and family impact. For all these models except one, the association between ADHD family status and impact on the family lost significance when EMF impairment was a covariate (all *p'*s > 0.05). The exception was the model testing for the impact on parental stress due to conflicts among siblings in the early morning (*p* = 0.04). To determine if the severity of EMF impairments associated with ADHD was predictive of impact on the family, we conducted regression analyses limited to the ADHD families that predicted the family impact variables from the severity of the ADHD child's EMF impairments. We found significant correlations between the EMF impairment severity of the ADHD child and all the family impact variables (all *p'*s < 0.001; correlations ranging from 0.44 to 0.68).

## Discussion

Although the ADHD children we surveyed had been treated with stimulant medications as their primary ADHD medication for at least 3 months, they still had elevated levels of EMF impairments compared with controls. This shows that ADHD children who express some ADHD symptoms in the morning are at risk for EMF impairments. These impairments diminished the emotional well-being of parents, interfered with the parental early morning routine, and increased the level of conflict among siblings and between caregiver and their spouse/partner. These findings confirm the survey findings reported by Sallee (2015), who also studied stimulant-treated youth with ADHD.

The impact of EMF impairments on the family was substantial. The parents in ADHD families were highly likely to report feeling adverse emotions, more conflict with spouses/partners, and greater disruption of the parental morning routine. The EMF impairments of ADHD children were also associated with higher levels of conflict with siblings and disruption of their morning routines. These findings are consistent with prior work indicating that families of patients with ADHD experience elevated levels of distress that impact family functioning (Whalen et al. 2006). Of particular concern, almost half of the parents of youth with ADHD expressed concern about the safety of their child and that of their other children due to the EMF impairments related to the child with ADHD. This finding is especially notable for clinicians given that children with ADHD are at significantly higher risk for a variety of types of accidents (Swensen et al. 2004) and the injury rates from accidents are reduced by medication treatment (Dalsgaard et al. 2015).

Consistent with our hypothesis, mediation analyses showed that the differences in family functioning between the families with and without ADHD were mediated (or could be accounted for) by the severity of EMF impairments in the index child. This finding, to some extent, reflects that nature of the questions about family impact, which asked about the effect of EMF impairments on family functioning (see Supplementary Data; Supplementary Data are available online at www.liebertpub.com/cap). Thus, the mediation analyses confirm that parents were responding as requested, that is, they were reporting problems in the family caused by the EMF impairments of the index child. These findings show that the adverse family functioning results were not simply due to the presence of ADHD in the child, but were, in fact, associated with the index child's EMF impairments. As further support for this idea, within the ADHD families, we found significant correlations between the EMF impairment severity of the ADHD child and all the family impact variables.

Our conclusions are tempered by several methodological limitations. We used an online survey to collect data rather than in person, structured interviews. The use of the former may have decreased the sensitivity of our assessments. It is, however, unlikely that the use of online methodology would have created spurious findings. Our survey had not previously been tested for either reliability or validity. Low reliability and validity would have added noise to the analyses and made it difficult to find statistical significance. Thus, negative findings should be interpreted with caution. In contrast, low reliability and validity would not explain the pattern of significant differences we found across many measures. Our design does not allow us to conclude if EMF impairments were due to delayed onset of stimulant effects or to underdosing or overall partial response.

We did not confirm the parental reports of their children's ADHD diagnoses and only collected EMF data from one parent. That means that some findings could be accounted for by method variance, that is, the parents may not have been able to discriminate EMF problems from global impairment and safety concerns. Without behavioral data or multiple respondents, we cannot with certainty separate child dysfunction from parental concern. Thus, using multiple respondents would have been ideal. Despite these concerns, single respondent surveys have a strong precedent for survey research, for example, it has been used by the Center for Disease Control (Visser et al. 2014). Moreover, using one parent to provide information about the psychiatric and functional status of children has a strong precedent in prior literature.

Because we only recruited families having children with ADHD who were stimulant treated, our findings may not generalize to families that have children with ADHD who are untreated or who are treated through other modalities. Like other surveys, our data are restricted to parent retrospective reports, not observations of actual behaviors. To most accurately assess the effects of ADHD on EMF impairments, the best design would be to include behavioral samples at early morning and at other times of the day and to evaluate whether our findings are specific to EMF or are, perhaps, simply a reflection of functional impairments throughout the day. Although the panel from which respondents were selected was representative of the U.S. population, given the low response rate, it is possible that our sample is biased in unknown ways. Thus, we cannot be sure to which populations our results will generalize. Our population cannot be biased, however, with regard to EMF because the email used to recruit respondents did not indicate a specific interest in ADHD children and adolescents with “problems in the morning” or “EMF Impairment” (or similar wording). Further evidence for lack of bias regarding EMF impairment is the fact that our 77% rate of caregiver-reported moderate-to-severe EMF impairment severity is similar to the rate of 76% reported by Sallee (2015).

## Conclusions

Within the constraints of these limitations, our findings show that the primary caregivers of stimulant-treated children and adolescents with ADHD report that inadequately controlled early morning ADHD symptoms and EMF impairments persist despite treatment. EMF impairments exert a pervasive and significantly negative emotional and functional burden, not only on the primary caregiver but also on the spouse/partner and siblings. This work, especially when considered in the context of similar findings by Sallee (2015), suggests that adequate ADHD symptom control during the early morning period may be an unmet need for school-age children with ADHD being treated with stimulant medications. More work is needed to confirm this finding, and to determine the degree to which symptom control at other times of day is also an unmet need.

## Clinical Significance

What are the clinical implications of the fact that stimulant-treated children show evidence of EMF impairments that impact their family? One approach is seen in the data presented. About half the parents indicated that they had woken up their child with ADHD earlier than normal to administer ADHD medication and then let them go back to sleep, so that the medication would provide control in the early morning. Most who used this strategy said it had a very positive or somewhat positive effect. Thus, this is an option clinicians could communicate to parents. The early morning routine also provides a well-defined target for behavioral family therapy or, for adolescents, cognitive behavior therapy. Using organizational charts and reinforcing clearly defined early morning behaviors could alleviate many of these problems. This suggests that psychosocial treatment programs should develop modules aimed at EMF impairments.

## Supplementary Material

Supplemental data

## Appendix

### Lightspeed GMI US Panel Demographic Profile (N = 1,269,000)

The profile of Lightspeed GMI US Panel participants overall reflects the demographic makeup of the United States. Key panel profile characteristics are shown below.

**Table:**

	*%*
Gender	
Male	31
Female	69
Household income	
Less than $25K	37
$25K–$49.9K	26
$50K–$74.9K	16
$75K–$99K	10
$100K+	12
Age	
18–24	19
25–34	28
35–44	21
45–54	17
55+	15
Region	
Midwest	21
Northeast	15
South	43
West	21
Race/ethnicity	
White/Caucasian	62
Black/African American	17
Hispanic/Latino	12
Other	9
Employment status	
House wife/house husband	8
Permanent full-time employment	26
Permanent part-time employment	8
Unpaid employment (e.g., volunteer work)/full-time care of family member	1
Retired	3
Self-employed/freelance	6
Student, in school or apprenticeship	6
Temporary, seasonal, or occasional work	2
Unable to work/disabled	5
Without work or currently not working, and looking for work	10
No answer	25
Education	
Grade school	1
Some high school	5
Graduated high school or GED	19
Some college—no degree	20
Graduated college—associate's degree	7
Graduated college—bachelor's degree	12
Postgraduate degree—MS, MA, MBA, MD, DVM, DDS, etc.	5
Technical school/vocational training	5
Doctorate—PhD	1
No answer	25
