# Supplementary material for: Early Morning Functional Impairments in Stimulant-Treated Children with Attention-Deficit/Hyperactivity Disorder Versus Controls: Impact on the Family
Source: J Child Adolesc Psychopharmacol. 2017 Oct 1;27(8):715–22. doi: 10.1089/cap.2016.0164 (PMC5651955; doi:10.1089/cap.2016.0164)
Supplement: Supplemental data [file Supp_Data.docx]

**Survey Questionnaire**

Estimated Length: 0 minutes

Method: Online

**INTRODUCTION**

Today we are conducting a marketing research study concerning health care for your family. Your opinions are important to us. Please continue with the survey.

**SCREENER**

1. **Are you the primary caregiver for one or more children between the ages of 6 and 17**?

Yes -1 **(continue)**

No/Prefer not to answer -2 **(terminate and tally)**

1. **Is one or more of the children between 6 and 17 years of age currently taking at least one medication for the treatment of ADHD**?

Yes -1 **(go to Q42)**

No -2 **(continue)**

Prefer not to answer -3 **(terminate and tally)**

**PARENTS OF NON-ADHD CHILDREN SECTION (Q3 – Q41)**

1. **For how many children between the ages of 6-17 in your household, are you the primary caregiver**?

**1 _______** -1

**2 _______** -2

**3 _______** -3

**4 or more ____** -4

**PROGRAMMER: parents and primary care givers who have more than 1 child AT Q3 – SHOW TEXT BELOW. IF ONLY 1 CHILD AT Q3, SKIP TEXT.**

**Instructions for parents and primary caregivers who have more than 1 child**

**If you have more than one child ages 6-17, please answer all of the following questions based on the child whose birthday is next**.

1. **Is that child**?

Male -1

Female -2

1. **What is that child’s age?** ________
2. **What is your relationship to that child? Are you the child’s**:

NOTE TO PROGRAMMER: For each of the 2 age groupings for the children, at least 65% of respondents must be a mother, father, step-mother or step-father.

Mother -1

Father -2

Step-mother -3

Step-father -4

Other primary caregiver -5

1. **Please mark all of the following conditions (if any), for which that child has been diagnosed**.

Oppositional Defiance Disorder -1

Conduct Disorder -2

Bipolar Disorder -3

Depression -4

Insomnia/Sleep Disorders -5

Tourette’s Disorder -6

Anxiety -7

Learning Disability -8

Developmental Disorder -9

None of these -10

**If yes to any conditions in Q7 - TERMINATE**

For these next questions**, impairment** refers to the child’s ability to successfully complete their Early Morning Routine at an age-appropriate level and without excessive parent supervision. This can also include the impact of their impairment on others.

**“Early Morning Routine”** refers to the time “from the moment the child awakens to the time they leave for school”.

1. ***On a scale from 1 to 10, where 1 means “Mildly Impaired” and 10 means “Very Severely Impaired”, how severe is that child’s functional impairment (or difficulty to function) during the early morning routine*?**

**IMPAIRMENT OF FUNCTIONING DURING THE EARLY MORNING (before school)**

| **Time of Day** | **NO Impairment** | **MILD Impairment** | | | **MODERATE Impairment** | | | | **SEVERE Impairment** | | |
| --- | --- | --- | --- | --- | --- | --- | --- | --- | --- | --- | --- |
| Early Morning Routine | 0 | 1 | 2 | 3 | 4 | 5 | 6 | 7 | 8 | 9 | 10 |

1. **During the Early Morning Routine, *(on the days when school is in session)*** **which of the following unwanted behaviors and impairments in functioning (if any) appear *frequently*** during this specific time period?

(Please check all that apply.)

| Unwanted Behaviors and Functional Impairments | Occur frequently during the Early Morning Routine |
| --- | --- |
| Impulsive – Acts without Thinking | -1 |
| Easily Distracted - Fails to finish things | -2 |
| Time Awareness – taking too long with morning routines | -3 |
| Argues a lot | -4 |
| Doesn’t follow directions | -5 |
| Procrastinates - waiting until the last moment to get out of bed, complete morning tasks | -6 |
| Misplaces or loses necessary Items (homework, lunch, backpack) | -7 |
| Too Dependent - unable to perform morning tasks by themselves such as dressing, hygiene, eating breakfast | -8 |
| Problems getting to school - misses the bus | -9 |
| Acts aggressively towards siblings or parents | -10 |
| `NONE OF THESE BEHAVIORS APPEAR FREQUENTLY | -11 |

1. **Considering your previous answers for the Early Morning Routine time period** (before school), when your child’s ability to function is impaired, **please fill in the box~~es~~ below by indicating the average number of school days each week that your child’s Early Morning Routine is impaired**.

| Average Number of School Days per Week That My Child’s Early Morning Routine (before school) is Impaired | 0 1 2 3 4 5 |
| --- | --- |

Researchers have often asked parents of children to describe how they may feel and may react to their child’s unwanted behaviors. Some of the feelings and reactions reported by parents are listed in the table immediately below.

1. **During the Early Morning Routine** when unwanted behaviors result**, which (if any) of the following describe your feelings or reactions *and their frequency of occurrence*** to your child’s unwanted behaviors and associated **impairments in Early Morning functioning? (Please indicate your estimate of the frequency of these feelings**.)

| How I may feel and react to my child’s unwanted behaviors during the Early Morning Routine... | | Never Occurs | Rarely Occurs | Sometimes Occurs | Often  Occurs |
| --- | --- | --- | --- | --- | --- |
| *Enjoy being with my child less...* | -1 | | -2 | -3 | -4 |
| *Feeling inadequate as a parent...* | 1 | | -2 | -3 | -4 |
| *Feeling overwhelmed and exhausted...* | **-1** | | -2 |  | -4 |
| *Raising my voice more than I want...* | -1 | | -2 | -3 | -4 |
| *Punishing more and praising less...* | -1 | | -2 | -3 | -4 |
| *Feeling frustrated that my child consumes all my time...* | -1 | | -2 | -3 | -4 |
| *Feeling guilty that my other children do not get adequate time from me....* | -1 | | -2 | -3 | -4 |
| *Feeling constantly stressed...* | -1 | | -2 | -3 | -4 |

1. **During the Early Morning Routine** when unwanted child behaviors result**, how would you describe the impact of these behaviors on your overall relationship with your child?**

Very Positive Impact on my overall relationship with my child -1

Somewhat Positive Impact on my overall relationship with my child -2

No Impact on my overall relationship with my child -3

Somewhat Negative Effect on my overall relationship with my child -4

Very Negative Effect on my overall relationship with my child -5

No such behavior -6

1. **How often** does your child’s behavior during the early morning routine (before school) **cause you concern** f**or their safety and well-being *in the home***?

Very Often [ ]

Often [ ]

Sometimes [ ]

Rarely [ ]

Never [ ]

1. **How often** does your child’s behavior during early the morning routine (before school) **cause you concern for their safety and well-being *outside the home***, such as in the neighborhood, at the bus stop, on the school bus, walking or riding their bike to school, or driving to school if they have a license?

Very Often [ ]

Often [ ]

Sometimes [ ]

Rarely [ ]

Never [ ]

1. **How often** does your child’s behavior during the early morning routine (before school) **cause you concern for the safety and well-being of their siblings *in the home***?

Very Often [ ]

Often [ ]

Sometimes [ ]

Rarely [ ]

Never [ ]

No Siblings [ ] **PROGRAMMER – SKIP TO Q17**

1. **How often** does your child’s behavior during the early morning routine (before school) **cause you concern for the** **safety and well-being of their** **siblings *outside the home***?

Very Often [ ]

Often [ ]

Sometimes [ ]

Rarely [ ]

Never [ ]

**PROGRAMMER: IF “NO SPOUSE/PARTNER” PICKED AT Q17, SKIP Q18, Q21, Q22, Q25, Q26, Q33, Q34**

| 1. **To what extent** does your child’s behavior during the early morning routine (before school) **contribute to conflict *with* *your spouse/partner*? PROGRAMMER: IF “DOES NOT APPLY” PICKED, SKIP TO Q19** | | | | | | | **18. How often does this conflict occur?** | | | | | |
| --- | --- | --- | --- | --- | --- | --- | --- | --- | --- | --- | --- | --- |
| Very Significantly | Significantly | Somewhat Significantly | Not too Significantly | Not at All Significantly | Does Not Apply | No Spouse/Partner | Very Often | Often | Sometimes | Rarely | Never | ~~No Spouse/Partner~~ |
| [ ] | [ ] | [ ] | [ ] | [ ] | [ ] | [ ] | [ ] | [ ] | [ ] | [ ] | [ ] | ~~[ ]~~ |

| **19. To what extent** does your child’s behavior during the early morning routine (before school) **contribute to disruption *of your own* preparations to get ready** for work or for your own daily obligations? **PROGRAMMER: IF “DOES NOT APPLY” PICKED, SKIP TO Q21** | | | | | | **20. How often does this conflict occur?** | | | | |
| --- | --- | --- | --- | --- | --- | --- | --- | --- | --- | --- |
| Very Significantly | Significantly | Somewhat Significantly | Not too Significantly | Not at All Significantly | Does Not Apply | Very Often | Often | Sometimes | Rarely | Never |
| [ ] | [ ] | [ ] | [ ] | [ ] | [ ] | [ ] | [ ] | [ ] | [ ] | [ ] |

| **21. To what extent** does your child’s behavior during the early morning routine (before school) **contribute to disruption of *your spouse/partner’s* preparations to get ready** for work or their own daily obligations? **PROGRAMMER: IF “DOES NOT APPLY” PICKED, SKIP TO Q23** | | | | | | | **22. How often does this conflict occur?** | | | | | |
| --- | --- | --- | --- | --- | --- | --- | --- | --- | --- | --- | --- | --- |
| Very Significantly | Significantly | Somewhat Significantly | Not too Significantly | Not at All Significantly | Does Not Apply | ~~No Spouse/Partner~~ | Very Often | Often | Sometimes | Rarely | Never | ~~No Spouse/Partner~~ |
| [ ] | [ ] | [ ] | [ ] | [ ] | [ ] | ~~[ ]~~ | [ ] | [ ] | [ ] | [ ] | [ ] | ~~[ ]~~ |

| **23. To what extent** does your child’s behavior during the early morning routine (before school) **interfere with** ***your* getting to work or getting other obligations done *on time***? **PROGRAMMER: IF “DOES NOT APPLY” PICKED, SKIP TO Q25** | | | | | | **24. How often does this conflict occur?** | | | | |
| --- | --- | --- | --- | --- | --- | --- | --- | --- | --- | --- |
| Very Significantly | Significantly | Somewhat Significantly | Not too Significantly | Not at All Significantly | Does Not Apply | Very Often | Often | Sometimes | Rarely | Never |
| [ ] | [ ] | [ ] | [ ] | [ ] | [ ] | [ ] | [ ] | [ ] | [ ] | [ ] |

| **25. To what extent** does your child’s behavior during the early morning routine (before school) **interfere with *your spouse/partner* getting to work or getting other obligations done *on time***? **PROGRAMMER: IF “DOES NOT APPLY” PICKED, SKIP TO Q27** | | | | | | | **26. How often does this conflict occur?** | | | | | |
| --- | --- | --- | --- | --- | --- | --- | --- | --- | --- | --- | --- | --- |
| Very Significantly | Significantly | Somewhat Significantly | Not too Significantly | Not at All Significantly | Does Not Apply | ~~No Spouse/Partner~~ | Very Often | Often | Sometimes | Rarely | Never | ~~No Spouse/Partner~~ |
| [ ] | [ ] | [ ] | [ ] | [ ] | [ ] | ~~[ ]~~ | [ ] | [ ] | [ ] | [ ] | [ ] | ~~[ ]~~ |

| **27**. **To what extent** does your child’s behavior during the early morning routine (before school) **prevent *the child* from getting an adequate breakfast?** ? **PROGRAMMER: IF “DOES NOT APPLY” PICKED, SKIP TO Q29** | | | | | | **28. How often does this conflict occur?** | | | | |
| --- | --- | --- | --- | --- | --- | --- | --- | --- | --- | --- |
| Very Significantly | Significantly | Somewhat Significantly | Not too Significantly | Not at All Significantly | Does Not Apply | Very Often | Often | Sometimes | Rarely | Never |
| [ ] | [ ] | [ ] | [ ] | [ ] | [ ] | [ ] | [ ] | [ ] | [ ] | [ ] |

| **29. To what extent** does your child’s behavior during the early morning routine (before school) **disrupt breakfast time *among your family members*? PROGRAMMER: IF “DOES NOT APPLY” PICKED, SKIP TO Q31** | | | | | | **30. How often does this conflict occur?** | | | | |
| --- | --- | --- | --- | --- | --- | --- | --- | --- | --- | --- |
| Very Significantly | Significantly | Somewhat Significantly | Not too Significantly | Not at All Significantly | Does Not Apply | Very Often | Often | Sometimes | Rarely | Never |
| [ ] | [ ] | [ ] | [ ] | [ ] | [ ] | [ ] | [ ] | [ ] | [ ] | [ ] |

**PROGRAMMER: IF OR “NO SIBLINGS” PICKED, SKIP Q32, Q35, Q36, Q37, Q38 Q39, Q40 + Q41**

| **31.** **To what extent** does your child’s behavior during the early morning routine (before school) **contribute to conflict *with their siblings*? PROGRAMMER: IF “DOES NOT APPLY” PICKED, SKIP TO Q33** | | | | | | | **32. How often does this conflict occur?** | | | | | |
| --- | --- | --- | --- | --- | --- | --- | --- | --- | --- | --- | --- | --- |
| Very Significantly | Significantly | Somewhat Significantly | Not too Significantly | Not at All Significantly | Does Not Apply | No Siblings | Very Often | Often | Sometimes | Rarely | Never | ~~No Siblings~~ |
| [ ] | [ ] | [ ] | [ ] | [ ] | [ ] | [ ] | [ ] | [ ] | [ ] | [ ] | [ ] | ~~[ ]~~ |

| **33. To what extent** do these **conflicts between your child and *their siblings*** during the early morning routine (before school) **create conflict between you and *your spouse/partner*? PROGRAMMER: IF “DOES NOT APPLY” OR PICKED, SKIP TO Q35** | | | | | | | **34. How often does this conflict occur?** | | | | | |
| --- | --- | --- | --- | --- | --- | --- | --- | --- | --- | --- | --- | --- |
| Very Significantly | Significantly | Somewhat Significantly | Not too Significantly | Not at All Significantly | Does Not Apply | ~~No Spouse/Partner~~ | Very Often | Often | Sometimes | Rarely | Never | ~~No Spouse/Partner~~ |
| [ ] | [ ] | [ ] | [ ] | [ ] | [ ] | ~~[ ]~~ | [ ] | [ ] | [ ] | [ ] | [ ] | ~~[ ]~~ |

| **35. To what extent** does your child’s behavior during the early morning routine (before school) **disrupt the ability of *their siblings* to get ready for school or other morning obligations? PROGRAMMER: IF “DOES NOT APPLY” PICKED, SKIP TO Q37** | | | | | | | **36. How often does this conflict occur?** | | | | | |
| --- | --- | --- | --- | --- | --- | --- | --- | --- | --- | --- | --- | --- |
| Very Significantly | Significantly | Somewhat Significantly | Not too Significantly | Not at All Significantly | Does Not Apply | ~~No Siblings~~ | Very Often | Often | Sometimes | Rarely | Never | ~~No Siblings~~ |
| [ ] | [ ] | [ ] | [ ] | [ ] | [ ] | ~~[ ]~~ | [ ] | [ ] | [ ] | [ ] | [ ] | ~~[ ]~~ |

| **37. To what extent** does your child’s behavior during the early morning routine (before school) **disrupt the ability of *their siblings* to get to school *on time*? PROGRAMMER: IF “DOES NOT APPLY” PICKED, SKIP TO Q39** | | | | | | | **38. How often does this conflict occur?** | | | | | |
| --- | --- | --- | --- | --- | --- | --- | --- | --- | --- | --- | --- | --- |
| Very Significantly | Significantly | Somewhat Significantly | Not too Significantly | Not at All Significantly | Does Not Apply | ~~No Siblings~~ | Very Often | Often | Sometimes | Rarely | Never | ~~No Siblings~~ |
| [ ] | [ ] | [ ] | [ ] | [ ] | [ ] | ~~[ ]~~ | [ ] | [ ] | [ ] | [ ] | [ ] | ~~[ ]~~ |

| **39. To what extent** do these **conflicts between your child and *their sibling****s* during the early morning routine (before school) **create stress *for you* during morning routines? PROGRAMMER: IF “DOES NOT APPLY” PICKED, SKIP TO Q41** | | | | | | | **40. How often does this conflict occur?** | | | | | |
| --- | --- | --- | --- | --- | --- | --- | --- | --- | --- | --- | --- | --- |
| Very Significantly | Significantly | Very Significantly | Not too Significantly | Not at All Significantly | Does Not Apply | ~~No Siblings~~ | Very Often | Often | Sometimes | Rarely | Never | ~~No Siblings~~ |
| [ ] | [ ] | [ ] | [ ] | [ ] | [ ] | ~~[ ]~~ | [ ] | [ ] | [ ] | [ ] | [ ] | ~~[ ]~~ |

**PROGRAMMER: IF OR “NO SIBLINGS” PICKED AT Q31, SKIP Q41 AND GO TO CLOSE OF NON-ADHD SURVEY**

1. **How often** do your child’s ***siblings complain* to you about** the child’s **misbehavior or** **disruption of the siblings’ early morning routines?**

Very Often [ ]

Often [ ]

Sometimes [ ]

Rarely [ ]

Never [ ]

***Those are all of our questions today. Thank you for your participation.***

**PROGRAMMER: END OF NON-ADHD PARENT SURVEY**

**PARENTS OF ADHD CHILDREN SECTION (Q42 – Q103)**

1. **What is the age of the child being treated for ADHD for whom you are the caregiver? (If more than one child is being treated for ADHD, mark all ages that apply**.)

Yes No

1. to 12 years of age -1 -2

13 to 17 years of age -1 -2

**PLEASE ADD SEPARATE SCREEN THAT REQUIRES RESPONDENT TO CLICK CONTINUE AFTER READING**

**PROGRAMMER: SHOW TEXT BELOW.**

Several of the survey questions that follow will ask you about “your child with ADHD” or “your ADHD child”. **If you have more than one child diagnosed with and treated for ADHD**, there is no reason to be confused. **You should select your ADHD child who has the most severe ADHD symptoms and answer each of the following questions for that specific child whenever you see questions about “your ADHD child“.**

1. **Thinking about your ADHD child, please indicate the one ADHD medication that is primarily used to treat the child’s symptoms.** Please keep in mind that all responses will be kept confidential and will only be reported in aggregate.

| ADHD MEDICATION | *Check only one* |
| --- | --- |
| Concerta (brand) | -1 (continue) |
| Concerta (generic) | -2 (continue) |
| Focalin / Focalin XR | -3 (continue) |
| ADDERALL XR (brand) | -4 (continue) |
| ADDERALL XR (generic) | -5 (continue) |
| Adderall (brand) | -6 (continue) |
| Adderall (generic) | -7 (continue) |
| Vyvanse (brand) | -8 (continue) |
| Daytrana (brand) | -9 (continue) |
| Ritalin (brand) | -10 (continue) |
| Ritalin LA (brand) | -11 (continue) |
| Ritalin (generic) | -12 (continue) |
| Metadate CD (brand) | -13 (continue) |
| Metadate CD (generic) | -14 (continue) |
| Quillivant XR (brand) | -15 (continue) |
| Aptensio XR (brand) | -16 (continue) |
| Evekeo (brand) | -17 (continue) |
| Zenzedi (brand) | -18 (continue) |
| Intuniv (brand) | -19 (terminate, tally) |
| Intuniv (generic) | -20 (terminate, tally) |
| Strattera (brand) | -21 (terminate, tally) |
| Kapvay (brand) | -22 (terminate, tally) |
| Kapvay (generic) | -23 (terminate, tally) |
| OTHER: (Specify)_______________________ | -24 (terminate, tally) |

1. **For how long has your ADHD child been taking their primary ADHD medication**?

Less than 3 months -1 (terminate and tally)

Between 3 and 6 months -2 (continue)

Between 6 months and 1 year -3 (continue)

Between 1 and 2 years -4 (continue)

More than 2 years -5 (continue)

1. **Thinking about the days when school is in session and when your ADHD child is taking his current ADHD medication*,* please rate the overall presence of any ADHD symptoms throughout the entire day on a scale from 1 to 10**, where 1 means “No ADHD symptoms exist while on Medication” and 10 means “Significant ADHD Symptoms Exist while on Medication”.

**No ADHD Symptoms Some ADHD Symptoms Significant ADHD Symptoms**

1 2 3 4 5 6 7 8 9 10

**PROGRAMMER: IF RESPONSE TO Q45 IS 1, TERMINATE AND TALLY**

1. **Thinking about the days when school is in session.** **For each of the times of day below that your ADHD child’s symptoms are not well-managed, please rate the typical severity of any ADHD symptoms during that time-period on a scale from 1 to 10,** where 1 means “No ADHD symptoms exist on Medication at this time of day” and 10 means “Significant ADHD Symptoms Exist while on Medication at this time of day”.

| Time of Day that ADHD Symptoms are NOT as Well-Managed | Typical Severity of ADHD Symptoms |
| --- | --- |
| Early Morning Routine  (definition: *from the moment the child awakens to the time they leave for school)* | **No ADHD Symptoms Some ADHD Symptoms Significant ADHD Symptoms**  1 2 3 4 5 6 7 8 9 10 |
| During School Day | **No ADHD Symptoms Some ADHD Symptoms Significant ADHD Symptoms**  1 2 3 4 5 6 7 8 9 10 |
| Afternoon Homework Time | **No ADHD Symptoms Some ADHD Symptoms Significant ADHD Symptoms**  1 2 3 4 5 6 7 8 9 10 |
| Dinner Time | **No ADHD Symptoms Some ADHD Symptoms Significant ADHD Symptoms**  1 2 3 4 5 6 7 8 9 10 |
| Bed Time | **No ADHD Symptoms Some Symptoms**  **Significant Symptoms**  1 2 3 4 5 6 7 8 9 10 |

**PROGRAMMER: IF RESPONSE TO Q46 “EARLY MORNING ROUTINE” IS 1, TERMINATE AND TALLY. Respondents with Likert Rating of ≥ 2 in Q46 qualify to enter the full survey and complete all of the following questions (Q47-Q103)**

1. **What is your relationship to the child/children with ADHD? Are you the child’s**:

**NOTE TO PROGRAMMER: For each of the 2 age groupings for the children, at least 65% of respondents must be a mother, father, step-mother or step-father.**

Mother -1

Father -2

Step-mother -3

Step-father -4

Other primary caregiver -5

1. **Within your ADHD child’s household, how many children are there under the age of 18?**

_____________

1. **Thinking of all the children in that household, what is the age and gender of each? (RECORD**)

**Allow response for ages less than 6**

**AGE GENDER**  **MALE FEMALE**

Child #1 __________ -1 -2

Child #2 __________ -1 -2

Child #3 __________ -1 -2

Child #4 __________ -1 -2

Child #5 __________ -1 -2

Child #6 __________ -1 -2

Child #7 __________ -1 -2

**TOTAL NUMBER OF CHILDREN ________**

1. **In total, how many of the children are taking medication for ADHD**? _________

***The following questions are asked regarding the child you care for who has the most severe symptoms of ADHD.***

1. **Is your ADHD child**:

Male -1

Female -2

1. **What is your ADHD child’s age?** ________
2. **How old was your ADHD child when [GENDER AT Q51] was diagnosed with ADHD?** ________
3. **How old was your ADHD child when [GENDER AT Q51] first began taking medication for ADHD?**  ________
4. **Is your child’s ADHD primarily characterized by**:

Hyperactivity and Impulsivity -1

Inattention -2

Both Hyperactivity/Impulsivity and Inattention -3

I don’t know -4

1. **Prior to being diagnosed and treated with medication for ADHD, on a scale from 1 to 10, where 1 means “Not At All Severe” and 10 means “Very Severe”, *how severe were the child’s ADHD symptoms (throughout the entire day)?***

**Not At Severe Moderately Severe Very Severe**

1 2 3 4 5 6 7 8 9 10

1. **Please mark all of the following conditions (if any), for which your ADHD child has also been diagnosed.**

Oppositional Defiant Disorder -1

Conduct Disorder -2

Bipolar Disorder -3

Depression -4

Insomnia/Sleep Disorders -5

Tourette’s Disorder -6

Anxiety -7

Learning Disability -8

Developmental Disorder -9

Other (specify) ___________ 10

None -X

1. **On the days when school is in session*,* please *indicate how many times per day* your ADHD child takes their primary ADHD Medication?**

Once-a-Day -1

Twice-a-Day -2

Three times-a-day -3

More than 3-times per day -4

1. **On the days when school is session, *what time of the day* does your ADHD child usually take their *first dose* of their primary ADHD Medication?**

Before 5 a.m. ______ -1

Between 5 a.m. and 6 a.m. _____ -2

Between 6 a.m. and 7 a.m. ______ -3

Between 7 a.m. and 8 a.m. ______ -4

After 8 a.m. ______ -5

1. **During the school-year,** **please indicate** ***how many days per week*** your ADHD child **takes their primary ADHD Medication**?

1 day per week -1

2 days per week -2

3 days per week -3

4 days per week -4

5 days per week -5

6 days per week -6

7 days per week -7

1. **On the days when school is in session*,*** **does your ADHD child *also take another ADHD medication* to supplement their primary ADHD medication?**

___YES -1

___ NO -2 **– SKIP TO Q63**

1. **On the days when school is in session**, ***what time of* *day does your child take* their supplemental ADHD medication? Please mark all times that apply.**

| Early Morning - before school -1 |
| --- |
| At School - during the school day -2 |
| Afternoon - after school -3 |
| Evening – after dinner -4 |

**PROGRAMMER: Multiple answers to Q62 are acceptable**

**PROGRAMMER: parents and primary care givers who have more than 1 child diagnosed with ADHD AT Q50 – SHOW TEXT BELOW. IF ONLY 1 CHILD WITH ADHD AT Q50, SKIP TEXT.**

Several of the survey questions that follow will ask you about “your child’s ADHD symptoms, ADHD impairment and unwanted behaviors.” **If you have more than one child diagnosed with and treated for ADHD, there is no reason to be confused. You should select your ADHD child who has the most severe ADHD symptoms and answer each of the following questions for that specific child.**

1. **Considering your previous answer for the “Early Morning Routine” *(on the days when school is in session)* and when your child’s ADHD symptoms are not as well managed, on a scale from 1 to 10, where 1 means “Mildly Impaired” and 10 means “Very Severely Impaired”, how *severe is your child’s functional impairment (or difficulty to function) as a result of their ADHD symptoms during the early morning routine?***

*For this question, impairment refers to the child’s ability to successfully complete their Early Morning Routine at an age-appropriate level and without excessive parent supervision. This can also include the impact of their impairment on others.*

**IMPAIRMENT OF FUNCTIONING DURING THE EARLY MORNING (before school)**

| **Time of Day** | **MILD Impairment** | | | **MODERATE Impairment** | | | | **SEVERE Impairment** | | |
| --- | --- | --- | --- | --- | --- | --- | --- | --- | --- | --- |
| Early Morning Routine | 1 | 2 | 3 | 4 | 5 | 6 | 7 | 8 | 9 | 10 |

1. **During the Early Morning Routine, *(on the days when school is in session)*** *and* when your child’s ADHD symptoms are not well-managed**, which of the following unwanted behaviors and impairments in functioning appear *frequently*** during this specific time period?

(Please check all that apply.)

| Unwanted Behaviors and Functional Impairments | Occur frequently during the Early Morning Routine |
| --- | --- |
| Impulsive – Acts without Thinking | -1 |
| Easily Distracted - Fails to finish things | -2 |
| Time Awareness – taking too long with morning routines | -3 |
| Argues a lot | -4 |
| Doesn’t follow directions | -5 |
| Procrastinates - waiting until the last moment to get out of bed, complete morning tasks | -6 |
| Misplaces or loses necessary Items (homework, lunch, backpack) | -7 |
| Too Dependent - unable to perform morning tasks by themselves such as dressing, hygiene, eating breakfast | -8 |
| Problems getting to school - misses the bus | -9 |
| Acts aggressively towards siblings or parents | -10 |
| None of these | -11 |

1. **Considering your previous answers for the Early Morning Routine time period** (before school), when your ADHD child’s ability to function is impaired, **please fill in the box below by indicating the average number of school days each week that your child’s Early Morning Routine is impaired.**

| Average Number of School Days per Week That My Child’s Early Morning Routine (before school) is Impaired | 0 1 2 3 4 5 |
| --- | --- |

**Researchers have often asked parents of ADHD children to describe how they may feel and may react to their child’s unwanted behaviors that result from their inadequately managed ADHD symptoms. Some of the feelings and reactions reported by parents are listed in the table immediately below.**

1. **During the Early Morning Routine** when your child’s ADHD symptoms are not well-managed and unwanted behaviors result**, which (if any) of the following describe your feelings or reactions *and their frequency of occurrence*** to your child’s unwanted behaviors and associated **impairments in Early Morning functioning? (Please indicate your estimate of the frequency of these feelings.)**

| How I may feel and react to my ADHD child’s unwanted behaviors during the Early Morning Routine... | | Never Occurs | Rarely Occurs | Sometimes Occurs | Often  Occurs |
| --- | --- | --- | --- | --- | --- |
| Enjoy being with my child less... | -1 | | -2 | -3 | -4 |
| Feeling inadequate as a parent... | 1 | | -2 | -3 | -4 |
| Feeling overwhelmed and exhausted... | **-1** | | -2 | -3 | -4 |
| Raising my voice more than I want... | -1 | | -2 | -3 | -4 |
| Punishing more and praising less... | -1 | | -2 | -3 | -4 |
| Feeling frustrated that my child consumes all my time... | -1 | | -2 | -3 | -4 |
| Feeling guilty that my other children do not get adequate  time from me.... | -1 | | -2 | -3 | -4 |
| Feeling constantly stressed... | -1 | | -2 | -3 | -4 |

1. **During the Early Morning Routine** when your child’s ADHD symptoms are not well-managed and unwanted child behaviors result**, how would you describe the impact of these behaviors on your overall relationship with your child?**

Very Positive Impact on my overall relationship with my child -1

Somewhat Positive Impact on my overall relationship with my child -2

No Impact on my overall relationship with my child -3

Somewhat Negative Effect on my overall relationship with my child -4

Very Negative Effect on my overall relationship with my child -5

**If answer to Q67 is “Very or Somewhat Positive”, ask Q68, otherwise go the Q69**

1. **Why do you say that?**

_____________________________________________________________________

1. **How often** do your child’s inadequately controlled ADHD symptoms during the early morning routine (before school) **cause you concern** f**or their safety and well-being *in the home***?

Very Often [ ]

Often [ ]

Sometimes [ ]

Rarely [ ]

Never [ ]

1. **How often** do your child’s inadequately controlled ADHD symptoms during early the morning routine (before school) **cause you concern for their safety and well-being *outside the home***, such as in the neighborhood, at the bus stop, on the school bus, walking or riding their bike to school, or driving to school if they have a license?

Very Often [ ]

Often [ ]

Sometimes [ ]

Rarely [ ]

Never [ ]

1. **How often** do your child’s inadequately controlled ADHD symptoms during the early morning routine (before school) **cause you concern for the safety and well-being of their siblings *in the home***?

Very Often [ ]

Often [ ]

Sometimes [ ]

Rarely [ ]

Never [ ]

No Siblings [ ] **PROGRAMMER – SKIP TO Q73**

1. **How often** do your child’s inadequately controlled ADHD symptoms during the early morning routine (before school) **cause you concern for the** **safety and well-being of their** **siblings *outside the home***?

Very Often [ ]

Often [ ]

Sometimes [ ]

Rarely [ ]

Never [ ]

No Siblings [ ]

**PROGRAMMER: IF “NO SPOUSE/PARTNER” PICKED AT Q73, SKIP Q74, Q77, Q78, Q81, Q82, Q89, Q90**

| 73. **To what extent** does your child’s inadequately controlled ADHD symptoms during the early morning routine (before school) **contribute to conflict *with* *your spouse/partner***? | | | | | | 74. **How often does this conflict occur**? | | | | | |
| --- | --- | --- | --- | --- | --- | --- | --- | --- | --- | --- | --- |
| Very Significantly | Significantly | Somewhat Significantly | Not too Significantly | Not at All Significantly | No Spouse/Partner | Very Often | Often | Sometimes | Rarely | Never | ~~No Spouse/Partner~~ |
| [ ] | [ ] | [ ] | [ ] | [ ] | [ ] | [ ] | [ ] | [ ] | [ ] | [ ] | ~~[ ]~~ |

| 75. **To what extent** does your child’s inadequately controlled ADHD symptoms during the early morning routine (before school) **contribute to disruption *of your own* preparations to get ready** for work or for your own daily obligations? | | | | | 76. **How often does this conflict occur**? | | | | |
| --- | --- | --- | --- | --- | --- | --- | --- | --- | --- |
| Very Significantly | Significantly | Somewhat Significantly | Not too Significantly | Not at All Significantly | Very Often | Often | Sometimes | Rarely | Never |
| [ ] | [ ] | [ ] | [ ] | [ ] | [ ] | [ ] | [ ] | [ ] | [ ] |

| **77. To what extent** does your child’s inadequately controlled ADHD symptoms during the early morning routine (before school) **contribute to disruption of *your spouse/partner’s* preparations to get ready** for work or their own daily obligations? | | | | | | 78**. How often does this conflict occur**? | | | | | |
| --- | --- | --- | --- | --- | --- | --- | --- | --- | --- | --- | --- |
| Very Significantly | Significantly | Somewhat Significantly | Not too Significantly | Not at All Significantly | ~~No Spouse/Partner~~ | Very Often | Often | Rarely | Sometimes | Never | ~~No Spouse/Partner~~ |
| [ ] | [ ] | [ ] | [ ] | [ ] | ~~[ ]~~ | [ ] | [ ] | [ ] | [ ] | [ ] | ~~[ ]~~ |

| 79. **To what extent** does your child’s inadequately controlled ADHD symptoms during the early morning routine (before school) **interfere with** ***your* getting to work or getting other obligations done *on time***? | | | | | 80. **How often does this conflict occur**? | | | | |
| --- | --- | --- | --- | --- | --- | --- | --- | --- | --- |
| Very Significantly | Significantly | Somewhat Significantly | Not too Significantly | Not at All Significantly | Very Often | Often | Sometimes | Rarely | Never |
| [ ] | [ ] | [ ] | [ ] | [ ] | [ ] | [ ] | [ ] | [ ] | [ ] |

| 81. **To what extent** does your child’s inadequately controlled ADHD symptoms during the early morning routine (before school) **interfere with *your spouse/partner* getting to work or getting other obligations done *on time***? | | | | | | 82. **How often does this conflict occur?** | | | | | |
| --- | --- | --- | --- | --- | --- | --- | --- | --- | --- | --- | --- |
| Very Significantly | Significantly | Somewhat Significantly | Not too Significantly | Not at All Significantly | ~~No Spouse/Partner~~ | Very Often | Often | Sometimes | Rarely | Never | ~~No Spouse/Partner~~ |
| [ ] | [ ] | [ ] | [ ] | [ ] | ~~[ ]~~ | [ ] | [ ] | [ ] | [ ] | [ ] | ~~[ ]~~ |

| 83. **To what extent** does your child’s inadequately controlled ADHD symptoms during the early morning routine (before school) **prevent *the child* from getting an adequate breakfast?** | | | | | 84. **How often does this conflict occur?** | | | | |
| --- | --- | --- | --- | --- | --- | --- | --- | --- | --- |
| Very Significantly | Significantly | Somewhat Significantly | Not too Significantly | Not at All Significantly | Very Often | Often | Sometimes | Rarely | Never |
| [ ] | [ ] | [ ] | [ ] | [ ] | [ ] | [ ] | [ ] | [ ] | [ ] |

| 85. **To what extent** does your child’s inadequately controlled ADHD symptoms during the early morning routine (before school) **disrupt breakfast time *among your family members*?** | | | | | 86. **How often does this conflict occur?** | | | | |
| --- | --- | --- | --- | --- | --- | --- | --- | --- | --- |
| Very Significantly | Significantly | Somewhat Significantly | Not too Significantly | Not at All Significantly | Very Often | Often | Sometimes | Rarely | Never |
| [ ] | [ ] | [ ] | [ ] | [ ] | [ ] | [ ] | [ ] | [ ] | [ ] |

**PROGRAMMER: IF “NO SIBLING” PICKED AT Q87, SKIP Q88, Q91, Q92, Q93, Q94, Q95, Q96 + Q97**

| **87. To what extent** does your child’s inadequately controlled ADHD symptoms during the early morning routine (before school) **contribute to conflict *with their siblings*?** | | | | | | 88**. How often does this conflict occur?** | | | | | |
| --- | --- | --- | --- | --- | --- | --- | --- | --- | --- | --- | --- |
| Very Significantly | Significantly | Somewhat Significantly | Not too Significantly | Not at All Significantly | No Siblings | Very Often | Often | Sometimes | Rarely | Never | ~~No Siblings~~ |
| [ ] | [ ] | [ ] | [ ] | [ ] | [ ] | [ ] | [ ] | [ ] | [ ] | [ ] | ~~[ ]~~ |

| 89. **To what extent** do these **conflicts between your ADHD child and *their siblings*** during the early morning routine (before school) **create conflict between you and *your spouse/partner*?** | | | | | | 90. **How often does this conflict occur?** | | | | | |
| --- | --- | --- | --- | --- | --- | --- | --- | --- | --- | --- | --- |
| Very Significantly | Significantly | Somewhat Significantly | Not too Significantly | Not at All Significantly | ~~No Spouse/Partner~~ | Very Often | Often | Sometimes | Rarely | Never | ~~No Spouse/Partner~~ |
| [ ] | [ ] | [ ] | [ ] | [ ] | ~~[ ]~~ | [ ] | [ ] | [ ] | [ ] | [ ] | ~~[ ]~~ |

| 91. **To what extent** does your child’s inadequately controlled ADHD symptoms during the early morning routine (before school) **disrupt the ability of *their siblings* to get ready for school or other morning obligations?** | | | | | | 92. **How often does this conflict occur?** | | | | | |
| --- | --- | --- | --- | --- | --- | --- | --- | --- | --- | --- | --- |
| Very Significantly | Significantly | Somewhat Significantly | Not too Significantly | Not at All Significantly | ~~No Siblings~~ | Very Often | Often | Sometimes | Rarely | Never | ~~No Siblings~~ |
| [ ] | [ ] | [ ] | [ ] | [ ] | ~~[ ]~~ | [ ] | [ ] | [ ] | [ ] | [ ] | ~~[ ]~~ |

| 93**. To what extent** does your child’s inadequately controlled ADHD symptoms during the early morning routine (before school) **disrupt the ability of *their siblings* to get to school or work *on time*?** | | | | | | 94. **How often does this conflict occur?** | | | | | |
| --- | --- | --- | --- | --- | --- | --- | --- | --- | --- | --- | --- |
| Very Significantly | Significantly | Somewhat Significantly | Not too Significantly | Not at All Significantly | ~~No Siblings~~ | Very Often | Often | Sometimes | Rarely | Never | ~~No Siblings~~ |
| [ ] | [ ] | [ ] | [ ] | [ ] | ~~[ ]~~ | [ ] | [ ] | [ ] | [ ] | [ ] | ~~[ ]~~ |

| 95. **To what extent** do these **conflicts between your ADHD child and *their sibling****s* during the early morning routine (before school) **create stress *for you* during morning routines?** | | | | | | 96. **How often does this conflict occur?** | | | | | |
| --- | --- | --- | --- | --- | --- | --- | --- | --- | --- | --- | --- |
| Very Significantly | Significantly | Very Significantly | Not too Significantly | Not at All Significantly | ~~No Siblings~~ | Very Often | Often | Sometimes | Rarely | Never | ~~No Siblings~~ |
| [ ] | [ ] | [ ] | [ ] | [ ] | ~~[ ]~~ | [ ] | [ ] | [ ] | [ ] | [ ] | ~~[ ]~~ |

**PROGRAMMER: IF “NO SIBLING” PICKED AT Q87, SKIP Q97**

1. **How often** do your ADHD child’s ***siblings complain* to you about** the ADHD child’s **misbehavior or** **disruption of the siblings’ early morning routines?**

Very Often [ ]

Often [ ]

Sometimes [ ]

Rarely [ ]

Never [ ]

~~No Siblings [ ]~~

1. **How satisfied are you with your child’s current ADHD medication** with regard to its ability to provide **meaningful relief of their ADHD symptoms during the Early Morning Routine (before school)?**

Extremely Satisfied -1

Very Satisfied -2

Somewhat Satisfied -3

Not Very Satisfied -4

Not At All Satisfied -5

1. **Have you ever discussed your ADHD child’s impaired early morning functioning with the doctor?**

Yes, we have discussed it -1

No, we have not discussed it -2

**~~PROGRAMMER: If “YES” to Q99 – go to Q100; If “NO”, go to Q101~~**

1. **What is the medical specialty of the doctor that prescribes your child's ADHD medication?**

Child and Adolescent Psychiatrist _____ -1

Developmental and Behavioral Pediatrician _____ -2

Child Neurologist _____ -3

Pediatrician _____ -4

Nurse Practitioner or Physician Assistant _____ -5

General Practice or Family Practice _____ -6

Other Specialty (specify) __________ _____ -7

1. **Have you or another adult in the household ever woken your ADHD child up earlier than their normal waking time to administer your child’s ADHD medication, and then let them go back to sleep, so that the medication could provide more effective ADHD symptom control from the time your child awakens until the time they leave for school?**

Yes, have awakened -1

No, have not awakened early -2

**PROGRAMMER: If “YES” to Q101 – go to Q102; If “NO”, survey is completed**

1. **On the days when school is in session, on average, how many days per week do you wake up early to administer your child's ADHD medication?**

0 ____ -1

1 ____ -2

2 ____ -3

3 ____ -4

4 ____ -5

5 ____ -6

**PROGRAMMER: If “1 or more” to Q102 – go to Q103; If “0”, survey is completed**

1. What is the overall **impact on you**, if any, **of your having to wake your child up earlier than necessary** in the morning, in order to administer your child’s ADHD medication?

Very Positive Impact _____ -1

Somewhat Positive Impact _____ -2

Neither Positive nor Negative Impact _____ -3

Somewhat Negative Impact _____ -4

Very Negative Impact _____ -5

***Those are all of our questions today. Thank you for your participation.***
